# Lactate Oxidase Disrupts Lactate-Activated RAS and PI3K Oncogenic Signaling

**DOI:** 10.3390/cancers16162817

**Published:** 2024-08-10

**Authors:** Chandler R. Keller, Steve R. Martinez, Alexys Keltz, Michelle Chen, Weimin Li

**Affiliations:** 1 Department of Translational Medicine and Physiology, Elson S. Floyd College of Medicine, Washington State University, Spokane, WA 99202, USA; 2 Department of Surgery, The Everett Clinic, Part of Optum, Everett, WA 98201, USA; 3 Providence Regional Cancer Partnership, Providence Regional Medical Center, Everett, WA 98201, USA; 4 Department of Medical Education and Clinical Sciences, Elson S. Floyd College of Medicine, Washington State University, Spokane, WA 99202, USA; 5 Eastern Washington University, Cheney, WA 99004, USA; 6 Ferris High School, Spokane, WA 99223, USA

**Keywords:** lactate oxidase (LOX), lactate receptor HCAR1, RAS/PI3K signaling, breast cancer (BCa), tumor microenvironment (TME)

## Abstract

**Simple Summary:**

The Aerococcus viridans bacterial lactate oxidase (LOX) is an enzyme absent in mammalian cells. LOX catalyzes lactate to pyruvate and hydrogen peroxide (H_2_O_2_), a function that was recently used for tumor microenvironment (TME) lactate removal and H_2_O_2_ production to induce cancer cell death. However, the functions of TME lactate other than being a metabolic intermediate or an energy source and the mechanism of LOX disruption of lactate-mediated cancer cell survival remain unknown. Our study aims at addressing these important cancer-related biological questions. We identified a novel onco-signaling nexus initiated by TME lactate activation of its cellular receptor hydroxycarboxylic acid receptor 1 (HCAR1) and amplified by HCAR-associated proteins that mediate the RAS and the PI3K pathways. This signaling model could explain many key survival strategies exerted by RAS, PI3K, and their effectors for cancer cell adaptation to TME changes, especially in the cells’ use of lactate beyond an energy source.

**Abstract:**

LOX was recently shown to inhibit cancer cell proliferation and tumor growth. The mechanism of this inhibition, however, has been exclusively attributed to LOX depletion of TME lactate, a cancer cell energy source, and production of H_2_O_2_, an oxidative stressor. We report that TME lactate triggers the assembly of the lactate receptor hydroxycarboxylic acid receptor 1 (HCAR1)-associated protein complex, which includes GRB2, SOS1, KRAS, GAB1, and PI3K, for the activation of both the RAS and the PI3K oncogenic signaling pathways in breast cancer (BCa) cells. LOX treatment decreased the levels of the proteins in the protein complex via induction of their proteasomal degradation. In addition, LOX inhibited lactate-stimulated expression of the lactate transporters MCT1 and MCT4. Our data suggest that HCAR1 activation by lactate is crucial for the assembly and function of the RAS and PI3K signaling nexus. Shutting down lactate signaling by disrupting this nexus could be detrimental to cancer cells. HCAR1 is therefore a promising target for the control of the RAS and the PI3K signaling required for BCa progression. Thus, our study provides insights into lactate signaling regulation of cancer progression and extends our understanding of LOX’s functional mechanisms that are fundamental for exploring its therapeutic potential.

## 1. Introduction

Glycolytic cancer cells produce and secrete L-lactate (lactate) that can be used as a metabolic fuel by oxidative cancer cells in the same tumor, forming a symbiotic mechanism for cancer cell survival and tumor progression [1]. In human blood and tissues, lactate levels may reach 2 mM at rest and up to 30 mM after strong physical activity [2,3]. Similarly, tumor tissue lactate levels range from 5 to 40 mM [4,5,6], suggesting highly active metabolic production of lactate in tumors. Lactate is a major gluconeogenic precursor, a highly active signaling molecule with hormone-like properties [7,8,9], acts as a tumor tissue immune suppressor, and is a cancer progression promotor [6,10,11,12] associated with cancer immune escape, self-sufficient metabolism, cell migration and invasion, angiogenesis, metastasis, recurrence, and poor overall survival in cancer patients [6,8,11,13,14]. 

Cellular lactate is mainly converted from pyruvate by lactate dehydrogenase A (LDHA), exported via monocarboxylate transporter 4 (MCT4) and imported by MCT1 [15]. These lactate production and transport activities are essential components of the symbiotic TME for efficient energy supply and preservation of cellular glucose for sustained tumor growth. Preclinical trials of LDHA and MCT inhibitors have demonstrated promise [16,17], but so far no LDHA inhibitors have entered clinical trials or become clinically viable treatments [18]. This may be due to the functional roles of LDHA in non-neoplastic cells and the bidirectional and near-equilibrium catalytic nature of LDH in pyruvate and lactate conversions [19]. A couple of MCT inhibitors have entered pre-clinical phases [14,16,20], but only one (AZD3965 for MCT1) is in the clinical phase I/II trial for Burkitt and large B cell lymphoma, gastric, and prostate cancers [1,13]. Extracellular lactate can also promote malignancy by activating HCAR1 (also called G-protein-coupled receptor 81 or GPR81) [21,22,23]. Though HCAR1 signaling via cyclic adenosine monophosphate (cAMP) and protein kinase A (PKA) has been observed in adipocytes and neuronal cells [24,25], and HCAR1 was shown to mediate macrophage and monocyte inflammatory response via arrestin β-2, toll-like receptor 4 (TLR4), and NLRP3 inflammasome [26,27], lactate-stimulated HCAR1 signaling in cancer cells is poorly understood. No drugs targeting HCAR1 have been developed for cancer treatment [28]. A better understanding of HCAR1 activation and regulation in cancer cells may facilitate the development of novel anticancer therapeutics.

A growing strategy to treat cancer is targeting TME biomolecules [29], and lactate is an ideal target. Our previous work showed that lactate is a major metabolite of BCa cells grown on tumor ECM [30]. Additionally, LOX was able to impair the survival of BCa cells of all molecular subtypes [30]. In contrast, normal mammary epithelial cells were minimally affected by LOX [30]. LOX is a member of the flavoenzyme family that functions differently than the mammalian intracellular lactate monooxygenase or dehydrogenase. It catalyzes the flavin mononucleotide (FMN, or riboflavin-5′-monophosphate)-dependent oxidation of lactate to pyruvate and H_2_O_2_ [31,32]. Based on this catalytic model, the current understanding about LOX inhibition of tumor development emphasizes LOX depletion of TME lactate and H_2_O_2_ oxidative stress induction of cancer cell death. This has been supported by the observations that LOX could convert tumor-promoting M2 type macrophages to tumor-suppressing M1 type macrophages [33], and LOX inhibited animal tumor growth and killed cancer cells with or without the inclusion of metal ions or catalase in the treatment system to amplify the H_2_O_2_’s toxicity by producing hydroxyl radicals or increasing the number of tumor-suppressing cells in the TME [29,33,34,35,36]. 

In this study, utilizing LOX’s lactate-depleting property, we identified HCAR1 activation-relaying proteins that assembled in a complex harboring the RAS and PI3K oncoproteins in response to lactate stimulation of BCa cells. Additionally, LOX treatment induction of degradation of the HCAR1-associated proteins and inactivation of the RAS and PI3K signaling were revealed. Our study therefore has defined a lactate-HCAR1-RAS/PI3K signaling cascade in BCa cells that is sensitive to TME lactate depletion and potentially a key mechanism mediating TME lactate-supported BCa cell survival and growth. Targeting the highly active HCAR1 in cancer cells, upstream of the RAS/PI3K signaling, could be an anticancer approach worthy of further evaluation. 

## 2. Materials and Methods

### 2.1. Cell Culture

MDA-MB-231 and T47D BCa cells (ATCC) were grown in 1 × DMEM containing 10% fetal bovine serum (FBS) and 1% penicillin and streptomycin. Normal primary human mammary epithelial cells (HMEpiC, ScienCell Research Laboratories) were grown in Mammary Epithelial Cell Medium (ScienCell Research Laboratories). MCF10A cells (ATCC) were cultured in DMEM/F12 composed of 5% horse serum, 20 ng/mL EGF, 1% penicillin/streptomycin, 0.5 μg/mL hydrocortisone, 100 ng/mL cholera toxin, and 10 μg/mL human insulin. All cells were cultured in a 37 °C incubator supplied with 5% CO_2_. Sodium L-lactate (Sigma) at 10 mM or the indicated doses for the dose-response experiment was used to stimulate the cells. LOX (AG Scientific) and MG132 (Sigma) at the indicated concentrations were used to treat the cells.

### 2.2. Measuring Lactate Levels in Breast Epithelial Cell Organoid Culture Medium

5 × 10^3^ BCa cells or normal mammary epithelial cells were suspended in 2 µL of human breast tissue matrix gel (HB-TMG), spotted in HB-TMG-coated 96-well plates and allowed the gel to polymerize, and cultured in optimal media for 7 days. The cells formed tumoroid/organoid structures after 24 h of culturing. Three replicate cultures were prepared for each experimental condition. LOX (0.05 U/mL) was added to the culture media 24 and 72 h post-cell seeding. A Lactate Plus meter (Nova Biomedical) was used to measure the concentrations of lactate in the culture media following the manufacturer’s instructions.

### 2.3. Vector

The TagRFP-T-LOX expressing vector was constructed by subcloning the synthetic construct monomeric red fluorescent protein TagRFP-T [37] (GenBank Accession EU582019) and the LOX coding sequences (GenBank Accession D50611; ordered from GenScript), spaced by an 18-base pair nucleotide linker, into a pGEX-6P-1 vector (Supplementary Figure S1). The pGEX-6P-1-TagRFP-T-LOX vector was expressed in BL21 E. Coli for TagRFP-T-LOX fusion protein expression. The red fluorescent LOX protein was affinity purified using glutathione agarose resin, which can bind with the GST-tag linked with the TagRFP-T-LOX protein that was used in our experiments.

### 2.4. RFP-LOX Treatment of Cancer Cell Tumoroids or HMEpiC Organoids

BCa cells or HMEpiC resuspended in HB-TMG (50,000 cells in 1 μL of hydrogel) were spotted at the bottom of the wells of 96-well plates (one spot per well), forming tumoroids or organoids. After gel polymerization at 37 °C, 200 μL of culture medium was added to the wells. Sodium phosphate solution (diluent, 0.1 M) or TagRFP-T-LOX (0.05 U/mL) was added to the culture media 24 h and 72 h after seeding the tumoroids or organoids. Accumulation of the red-colored TagRFP-T-LOX in the tumoroids or organoids and the growth of the tumoroids/organoids were observed and imaged using fluorescence and bright field microscopy at the indicated time points over a 96-h time window after the initial LOX addition. 

### 2.5. Migration Assay

The wells of 96-well plates were coated with 5 μL of HB-TMG (2 mg/mL), which was polymerized in an incubator (37 °C, 5% CO_2_) and air-dried in a biosafety cabinet. HB-TMG (4 mg/mL) containing HMEpiC or BCa cells (400,000 cells in 1 μL) were spotted at the bottom of the hydrogel-coated wells and polymerized in the incubator, followed by carefully adding 300 μL of medium and cultured for 24 h. Diluent or LOX treatment was performed as before every other day after cell seeding for up to 144 h. Cell migration out of the spotted tumoroid on the HB-TMG matrix was imaged using brightfield microscopy. The images were analyzed with ImageJ software (1.53r). A circular border was drawn around the cell tumoroid image corresponding to the treatment start point during quantitative analysis. The percent increase in cell migration out of the circularized border regions was calculated and analyzed for differences between the experimental groups. 

### 2.6. Western Blot

Whole protein extracts from cultured cells were subjected to SDS-PAGE and Western blot for detection of specific protein expression in the cells following our established protocol [38]. The intensities of the protein bands were quantified using ImageJ software where necessary. Antibodies for HCAR (Abcepta, CA, USA), GRB2 (Santa Cruz Biotechnology, TX, USA), SOS1 (Proteintech, IL, USA), KRAS (Santa Cruz Biotechnology, TX, USA), GAB1 (Novus Biologicals, CO, USA), p85 (Proteintech, IL, USA), p110β (Cell Signaling Technology, MA, USA), p110α (Boster Bio, CA, USA), pERK1/2 (Santa Cruz Biotechnology, TX, USA), ERK1/2 (Santa Cruz Biotechnology, TX, USA), AKT (Cell Signaling Technology, MA, USA), pAKT^S473^ (Cell Signaling Technology, MA, USA), pAKT^T308^ (Cell Signaling Technology, MA, USA), MCT1 (Santa Cruz Biotechnology, TX, USA), MCT4 (Santa Cruz Biotechnology, TX, USA), and Actin (Thermo Scientific, MA, USA) were used in this study.

### 2.7. Protein Immunoprecipitation (IP)

Protein IP was performed as previously reported [39]. Briefly, the HCAR1 protein was immunoprecipitated with a specific antibody from 500 μg of total protein extracts of the cells cultured under different conditions. The HCAR1-associated proteins of interest were detected with Western blot. IgG alone was used as an IP control. 

### 2.8. Real-Time PCR 

This was performed as previously reported [38]. Briefly, the total RNA from the cells treated under the different experimental conditions were extracted and reversely transcribed to cDNA. The mRNA expression of the target genes were detected with a Bio-Rad CFX96 Real-Time thermal cycler. Single product amplification was confirmed by melting curve analysis. The target mRNA abundance was normalized to actin beta expression.

HCAR1 forward primer: CTCATTGTGGCCTTTGTGCT

HCAR1 reverse primer: GAATGTCCCCAAAAGCCCAG

KRAS forward primer: AGTGCCTTGACGATACAGCT

KRAS reverse primer: CCTCCCCAGTCCTCATGTAC

GRB2 forward primer: AGACGGCTTCATTCCCAAGA

GRB2 reverse primer: TGCTGCACATCGTTTCCAAA

SOS1 forward primer: CACCTCCTCCTCAAACACCT

SOS1 reverse primer: GTGTGTGTGCTCCCTTTTGT

GAB1 forward primer: ACCCAAACCTGTCCAGTGAA

GAB1 reverse primer: TTTGCTTACGTGGTGGTGTG

PIK3R1 (p85) forward primer: ACCACTACCGGAATGAATCTCT

PIK3R1 (p85) reverse primer: GGGATGTGCGGGTATATTCTTC

MCT1 forward primer: GCCCTGTGTTCCTCTGTACT

MCT1 reverse primer: CCAGCTTTCTCAAGGGATGC

MCT4 forward primer: AGCAGGTATCCTTGAGACGG

MCT4 reverse primer: GGCAAAGCAGATGGTGTAGG

ACTB forward primer: GGACTTCGAGCAAGAGATGG

ACTB reverse primer: AGCACTGTGTTGGCGTACAG

### 2.9. SiRNA Knockdown

BCa cells were seeded in 6-well culture plates and transfected with the indicated siRNAs using Lipofectamine 3000 (Thermo Fisher Scientific, MA, USA) following the manufacturer’s instructions. The cells were collected 48 h after transfection. Total cellular proteins were extracted for Western blot analysis of protein expression changes.

Scrambled siRNA: AGGUAGUGUAAUCGCCUUG

HCAR1 siRNA-1: GAUAGAGUGGUGACUUAGA

HCAR1 siRNA-2: CAAAUAGUUUCCAAAGCCA

GRB2 siRNA-1: CGAAGAAUGUGAUCAGAAC

GRB2 siRNA-2: UGAAUGAGCUGGUGGAUUA

KRAS siRNA-1: UGACGAUACAGCUAAUUCA

KRAS siRNA-2: GGACGAAUAUGAUCCAACA

PIK3CB siRNA-1: CCACTGGGATTTATATCCAGTTGGA

PIK3CB siRNA-2: GCTCCATACCTGTGGATTT

### 2.10. Statistics and Analysis 

The experiments involving quantitative analyses were conducted in three biological repeats, with each experimental condition having triplicate samples. The differences between the experimental groups were analyzed using one-way ANOVA and post hoc Tukey honestly significant difference tests for statistical significance, which were based on 95% confidence intervals. A *p*-value less than 0.05 was considered statistically significant. 

## 3. Results

### 3.1. LOX Depleted the Lactate Produced by BCa Cell Tumoroids Grown in Human Breast ECM 3D Cultures and Inhibited the Tumoroid Growth

**Figure 1:**
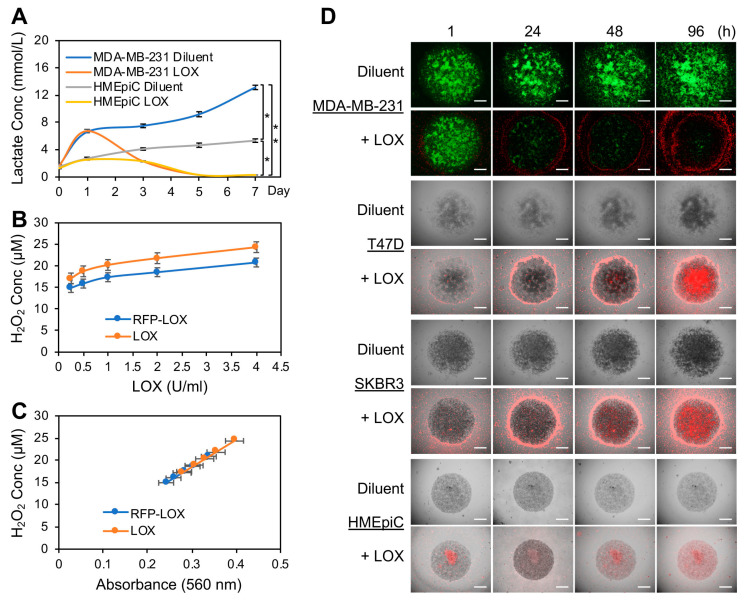
LOX depleted TME lactate secreted by BCa cell tumoroids and killed the tumoroid cells. (**A**) MDA-MB-231 or HMEpiC cells were grown as tumoroids or organoids in HB-TMG for 7 days. LOX (0.05 U/mL) was added to the cultures 24 and 72 h after cell seeding. The lactate levels in the culture media were measured with a lactate meter. Error bars, mean ± SD of 3 biological repeats. * *p* < 0.05; ** *p* < 0.001. (**B**) TagRFP-T-LOX and commercial LOX enzymatic activity comparison based on H_2_O_2_ production from the reactions of LOX at increasing concentrations and sodium lactate at 10 mM. (**C**) The H_2_O_2_ concentrations relative to the absorbance of the fluorescent signals produced in the B panel reactions. Error bars, Mean ± SD of 3 biological replicates. (**D**) Examination of TagRFP-T-LOX accumulation in BCa cell tumoroids and HMEpiC organoids. The indicated times were the imaging time points after LOX addition to the culture media. Scale bars, 200 μm.

Our previous work showed that lactate is a major metabolite secreted from proliferating BCa cells grown on pig breast tissue ECM [30]. We therefore speculated that the presence of LOX within the TME would remove proliferating cancer cell-generated lactate. To confirm this, we encapsulated MDA-MB-231 human BCa cells or normal primary human mammary epithelial cells (HMEpiC) in human breast tissue matrix gel (HB-TMG) [40] to form tumoroids or organoids in 96-well plates. HB-TMG, like pig or mouse breast tissue matrix gel (PB-TMG or MB-TMG), has ECM protein compositions and ECM microstructures like those of native ECM that serve as signaling ligands and physical supports, respectively, essential for mammary epithelial cells to display proper morphologies, membrane receptors, biological activities, and responses to drugs similar to those seen in native tissues, as we reported [30,40,41,42,43]. Therefore, HB-TMG provides an ideal tissue culture model for the observation of human breast epithelial cell biological phenotypes. The tumoroids or organoids were grown for 7 days, with 0.05 U/mL LOX solution or 0.1 M sodium phosphate solution (diluent) added on day 1 (24 h after cell seeding) and day 3 of the cultures. Lactate concentrations in the culture media were measured using a lactate meter 6 h after seeding the cells and at day 1, 3, 5, and 7 time points. Lactate levels in the diluent-treated MDA-MB-231 cell cultures increased quickly over time and reached about 13 mM while the HMEpiC culture lactate levels reached about 5 mM on day 7 (Figure 1A). LOX treatment quickly reduced MDA-MB-231 cells and gradually decreased HMEpiC culture lactate levels, respectively (Figure 1A). By day 5 of the cultures, the lactate concentrations in the culture media of both cell types were decreased to non-detectable levels (<0.3 mM). A similar lactate depletion effect was observed by others using LOX-loaded nanoparticles [44]. These observations indicate that LOX was able to deplete lactate in the TME when supplied or accumulated locally.

Since tumor edge regions contain fast-growing cancer cells, we speculated that those regions could function as reservoirs for lactate and hypothesized that clearing the locally produced lactate would inhibit tumor growth. To prove this, we engineered and affinity-purified a LOX protein fused with an enhanced red fluorescence protein TagRFP-T, as reported elsewhere [37]. The resulting TagRFP-T-LOX (Supplementary Figure S1) had enzymatic activity comparable to that of commercial LOX (AG Scientific; Figure 1B,C; no statistical differences) as assayed for their reactions with 10 mM sodium lactate to produce H_2_O_2_ (proportional to LOX activity) that was colorimetrically measured with the Amplex Red H_2_O_2_ Assay (Thermo Fisher Scientific, MA, USA). Using the TagRFP-T-LOX, we inspected lactate accumulation and its clearance by LOX in tumoroids encapsulated in HB-TMG, with LOX treatment at 24 h and 72 h after seeding the tumoroids as detailed in the methods. HMEpiC organoids were used along with the tumoroids for phenotypic comparison. TagRFP-T-LOX started accumulating around the growing edges of the MDA-MB-231, T47D, and SKBR3 tumoroids within 1 h after LOX addition to the culture media. TagRFP-T-LOX progressively penetrated and accumulated toward the crowded and almost non-expanding cancer cell population in the center of the tumoroids over time, accompanied by progressive cancer cell death, as demonstrated by the fading signals in the green fluorescence protein (GFP)-tagged MDA-MB-231 cells (Figure 1D, top). In contrast, TagRFP-T-LOX was only diffusely distributed, with accumulation in small areas, in the HMEpiC organoids formed by high-passage HMEpiC, which were slowly proliferating in tissue cultures (Figure 1D, bottom). These data indicate that LOX accumulates in the areas of fast-growing cells where lactate production is potentially higher than those of slow- or non-growing cells.

### 3.2. LOX Treatment Impaired Cancer Cell Migration on Native Breast ECM

**Figure 2:**
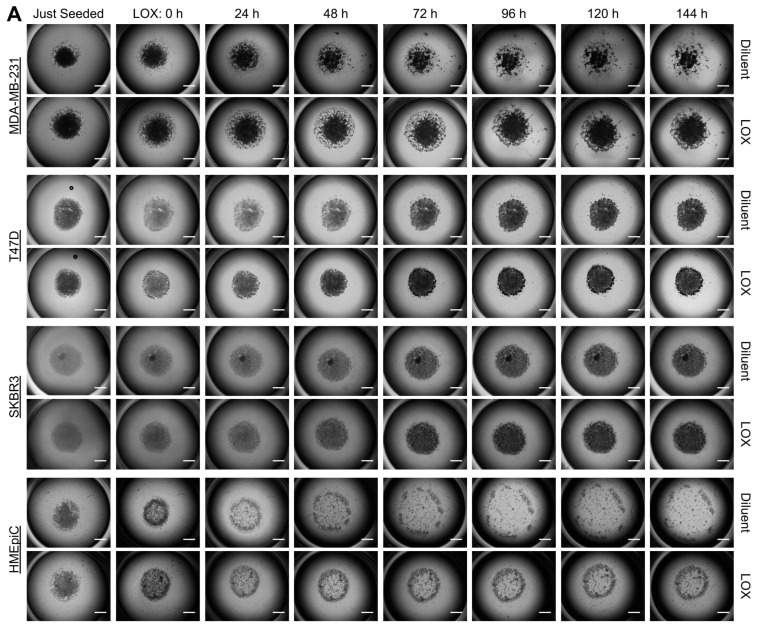

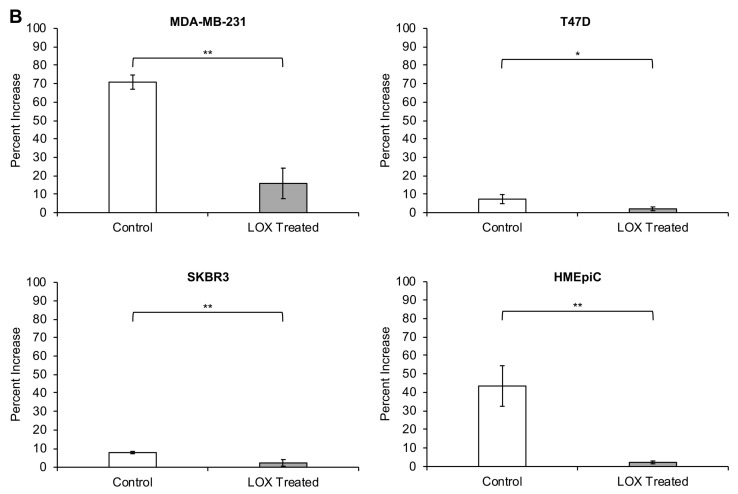
BCa cell migration on HB-TMG. (**A**) The migration of MDA-MB-231, T47D, or SKBR3 cells resuspended in HB-TMG, spotted at the bottom of HB-TMG-coated 96-well plates, and grown under optimal conditions were imaged with bright-field microscopy over time of cultures. HMEpiC cultures were used for comparisons. Scale bars, 1 mm. (**B**) Quantifications of the migration assays shown in (**A**). Error bars, Mean ± SD of three biological replicates. * *p* < 0.05; ** *p* < 0.01.

Since lactate within the TME is associated with cancer cell migration and metastasis [11,45], we anticipated that LOX removal of lactate in the tissue culture microenvironment would inhibit cancer cell migration. To test this under a biologically relevant condition, the wells of 96-well plates were coated with HB-TMG with an elastic modulus (2 mg/mL) corresponding to that of human breast tissue ECM as we reported before [40], followed by spotting MDA-MB-231, T47D, or SKBR3 BCa cell tumoroids or HMEpiC organoids suspended in stiffer HB-TMG, with an elastic modulus of 4 mg/mL corresponding to that of breast tumors, in the middle of the wells. The tumoroids or organoids were cultured under optimal conditions in the presence or absence of LOX (0.05 U/mL) or sodium phosphate diluent (0.1 M) treatment every other day 24 h after seeding. Cell migration out of the tumoroids/organoids into the surrounding matrix was imaged and measured (Figure 2). All the cell types tested exhibited migration on HB-TMG, with MDA-MB-231 cells migrating the fastest and T47D the slowest among the four groups (Figure 2A,B). LOX treatment significantly blocked the cell migration, with higher degrees of inhibition in faster-migrating cells than in slower-moving cells (Figure 2A,B). We also observed that MDA-MB-231 cells migrated in an evenly radial manner whereas the other types of BCa cells migrated unevenly and slowly in satellite clusters. It is interesting that HMEpiC cells migrated faster than T47D or SKBR3 cells on HB-TMGT and were sensitive to LOX treatment though their proliferation was slower than the BCa cells and not sensitive to LOX treatment as we previously reported [30]. These observations suggest that LOX inhibits mammary epithelial cell migration in the native tissue microenvironment.

### 3.3. Lactate Stimulated HCAR1 Association with the Mediators of the RAS and PI3K Signaling Pathways 

Studies of biological lactate regulation have primarily focused on its glycolytic generation, mitochondrial oxidation, and cellular transport. TME lactate signaling regulation of cancer progression remains largely unknown. The function of LOX’s effective depletion of TME lactate (Figure 1A) provides a perfect tool for identifying lactate-mediated signaling cascades essential for cancer cell survival and disease progression. Though lactate receptor HCAR1 signaling via cyclic adenosine monophosphate (cAMP) and protein kinase A (PKA) has been observed in neuronal cells [24], and HCAR1 was shown to mediate macrophage and monocyte inflammatory response via arrestin β-2, toll-like receptor 4 (TLR4), and NLRP3 inflammasome [26], lactate-stimulated HCAR1 signaling in cancer cells has not been defined. Since HCAR1 is a G-protein-coupled receptor (GPCR) and RAS can be activated by GPCR, we speculated that lactate activation of HCAR1 may induce RAS activation via activated HCAR1-associated proteins. Importantly, though RAS plays an essential role in human cancer initiation and progression [46,47,48], how lactate mediates RAS expression, activation, and signaling are unknown.

**Figure 3:**
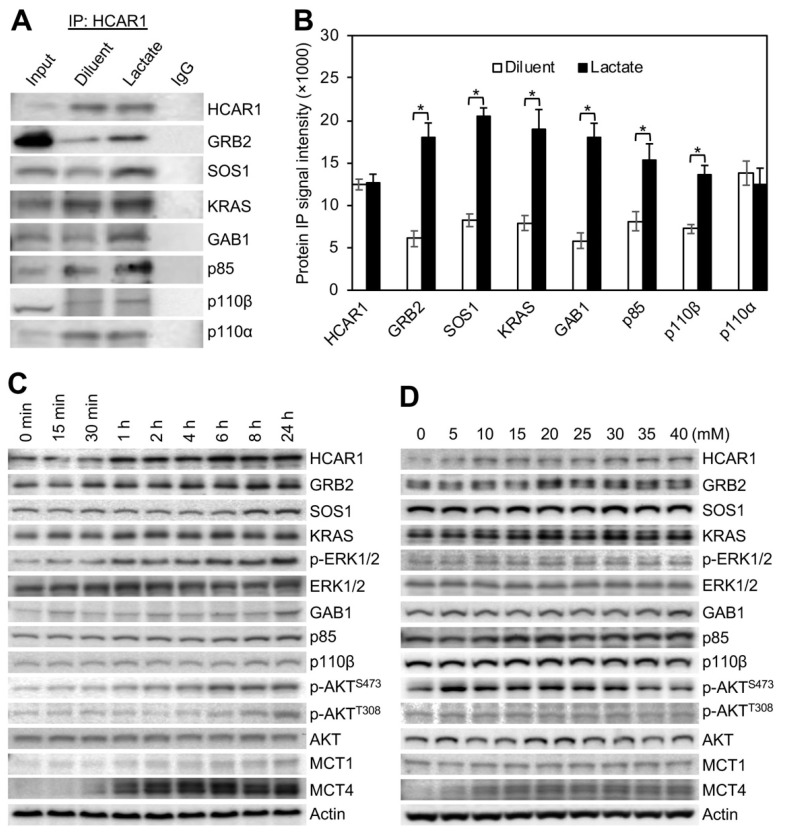
Lactate increased the association of RAS and PI3K signaling pathway proteins with HCAR1. (**A**) IP of HCAR1 in MDA-MB-231 cell total protein lysates revealed the association of HCAR1 with RAS and PI3K signaling activators that was enhanced by lactate stimulation for 24 h (Western blot). (**B**) Quantification of the signal intensities of the protein bands shown in lane-2 and lane-3 of figure panel A. Error bars, mean ± SD of 3 biological repeats. * *p* < 0.05. (**C**) Lactate stimulation (10 mM) time-dependently increased the expression of HCAR1, HCAR1-associated proteins in the RAS and PI3K signaling pathways, which were activated as indicated by ERK1/2 and AKT phosphorylation, and MCT1 and MCT4. (**D**) HCAR1-associated protein expression and RAS and PI3K pathway activation in response to increasing concentrations of lactate stimulation for 6 h.

To identify the HCAR1-associated proteins that activate RAS signaling in response to lactate stimulation, we immunoprecipitated (IP) HCAR1 with HCAR1-specific antibody in the total cell lysates of MDA-MB-231 cells treated with or without lactate and performed Western blot to detect common GPCR-associated proteins that are involved in cell growth regulation. We identified a set of proteins in the complex with HCAR1 that belong to two signaling cascades. These are growth factor receptor bound protein 2 (GRB2), son of sevenless homologue 1 (SOS1) and Kirsten rat sarcoma virus (KRAS) that are key mediators of the oncogenic RAS signaling pathway and GRB2 associated binding protein 1 (GAB1), the phosphatidylinositol 3-kinase (PI3K) regulatory subunit p85, and PI3K catalytic subunits p110α and p110β that are essential regulators of the PI3K signaling pathway (Figure 3A). The levels of these factors in the HCAR1 protein complex were increased after lactate stimulation, except p110α (Figure 3A,B). P110γ and p110δ were not detected in the complex with HCAR1 (data not shown). Similar IP data were obtained using the protein samples from the luminal A type T47D BCa cells (Supplementary Figure S2). Consistently, lactate stimulation time-dependently increased the expression of HCAR1 and its associated GRB2, SOS1, KRAS, GAB1, and p85 as well as MCT1 and MCT4 (Figure 3C), while lactate at increasing concentrations only augmented HCAR1, KRAS and, to a less extent, GRB2 levels in 6 h of treatment (Figure 3D; the time of treatment selection was based on the time-course experiment shown in Figure 3C). Activation of the RAS and PI3K signaling cascades by lactate stimulation was evidenced by AKT and ERK1/2 phosphorylation (Figure 3C,D).

### 3.4. LOX Treatment Abrogated Lactate-Stimulated HCAR1 and Its Associated Protein Expression as Well as the RAS and the PI3K Signaling 

**Figure 4:**
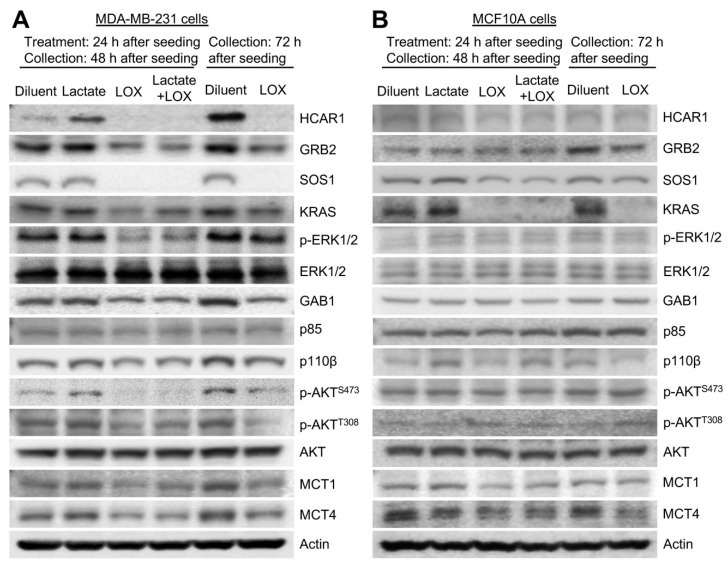
Lactate and LOX mediated the expression of HCAR1 and its associated RAS and PI3K pathway proteins in BCa cells differently than in normal mammary epithelial cells. (**A**) Lactate stimulated and LOX abrogated the expression of HCAR1 and its associated RAS and PI3K pathway proteins as well as the activation of the pathways. (**B**) Lactate and LOX treatment only unambiguously affected KRAS expression but not the activation of the RAS and PI3K pathways in MCF10A cells.

We next examined the effect of LOX depletion of TME lactate on the expression of HCAR1 and its associated signaling proteins. MDA-MB-231 BCa cells or normal MCF10A cells cultured under optimal conditions were treated with diluent (0.1 M sodium phosphate), sodium lactate (10 mM), LOX (0.05 U/mL), or lactate and LOX 24 h after cell seeding. The cells were collected 24 h, 48 h, or 72 h after the treatment and lysed to obtain total cellular proteins, which were subjected to Western blot analysis of the proteins of interest. Our results showed that both exogenous and BCa cell-generated lactate stimulated HCAR1, GRB2, SOS1, KRAS, GAB1, p110β, MCT1, and MCT4 expression in MDA-MB-231 cells (Figure 4A, lanes 2 and 5). LOX treatment decreased the HCAR1-associated proteins to either basal or below basal levels (Figure 4A, lanes 3, 4 and 6). Importantly, lactate-stimulated AKT and ERK1/2 phosphorylation were blocked by LOX treatment (Figure 4A, lanes 3, 4 and 6). Interestingly, exogenous lactate (Figure 4A, lane 2) failed to induce MCT1 expression above the basal levels after 24 h of treatment of the BCa cells, whereas a 72-h culture (Figure 4A, lane 5) substantially increased MCT1 levels, possibly due to increased cell-secretion of lactate into the culture medium. Still, LOX diminished both the basal and the extended culture-induced MCT1 expression (Figure 4A, lane 3 and lane 6). In contrast, exogenous lactate failed to increase HCAR1 and its associated protein levels, and extended culture at 72 h only increased GRB2 and KRAS levels in MCF10A cells (Figure 4B). LOX treatment reduced KRAS and, to a lesser extent, SOS1, MCT1, and MCT4 levels in the normal cells (Figure 4B). Neither lactate nor LOX seemed to have any impact on AKT or ERK1/2 phosphorylation in MCF10A cells. These data suggest that LOX treatment impaired the expression of BCa cell HCAR1 and its associated proteins involved in RAS and PI3K signaling as well as the lactate transporters. 

### 3.5. LOX Treatment Induced Proteasomal Degradation of BCa Cell HCAR1 and Its Associated Proteins as Well as the MCTs

**Figure 5:**
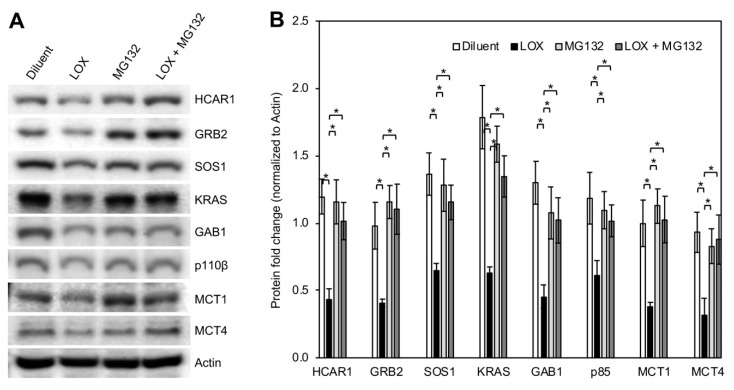
Proteasome inhibition blocked LOX-induced degradation of HCAR1, its associated proteins, and the MCTs. (**A**) Western blots of the protein expression after LOX or/and MG132 treatment for 24 h. Diluent, 0.1 M sodium phosphate buffer; lactate, 10 mM; LOX, 0.05 U/mL. (**B**) Quantification of the signal intensities of the protein bands shown in panel A. Error bars, mean ± SD of 3 biological repeats. * *p* < 0.05.

To define the mechanism of LOX-induced decrease of BCa cell HCAR1 and its associated proteins, we examined the expression of the proteins at degradation and transcription levels. Subconfluent MDA-MB-231 cells were treated with or without LOX (0.05 U/mL) in the presence or absence of proteasome inhibitor MG132 (5 μM) for 24 h and extracted for total proteins, which were subsequently used for Western blot detection of protein level changes. The results showed that MG132 blocked the LOX-induced decrease of HCAR1 and the associated proteins GRB2, SOS1, KRAS, GAB1, and p110β (Figure 5A,B). LOX downregulation of MCT1 and MCT4 protein levels were also abolished by MG132. These data indicate that LOX treatment triggered proteasomal degradation of HCAR1, the receptor-associated proteins, and the MCTs. The same effect was observed in T47D BCa cells (Supplementary Figure S3). Quantitative real-time PCR analysis of the mRNA expression of the LOX treatment-affected HCAR1 and its associated proteins in MDA-MB-231 cells showed that lactate stimulation only significantly increased HCAR1 and MCT1 gene expression in a LOX treatment-sensitive manner (Supplementary Figure S4). LOX treatment had no significant impact on the expression of the genes whose products are in the HCAR1 complex that are regulated by LOX at degradation levels. 

### 3.6. Downregulation of HCAR1 and Its-Associated Proteins Key for RAS and PI3K Signaling Interrupted Lactate-Stimulated Activation of the Two Pathways

**Figure 6:**
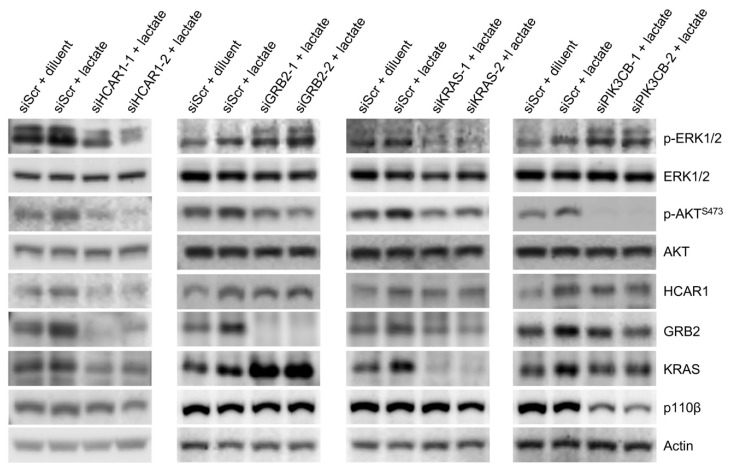
Knockdown of HCAR1 or its key associated proteins involved in RAS or PI3K signaling interrupted lactate-stimulated activation of the two pathways. Subconfluent MDA-MB-231 cells were knocked down for the indicated proteins with small interfering RNA (siRNA) and treated with 10 mM lactate for 24 h, followed by total protein extraction and Western blot detection for protein expression changes. Scr, scrambled RNA as non-target control. Two different siRNAs for each target gene were used in the experiment.

Since lactate stimulation not only increased HCAR1 levels (Figure 3C,D and Figure 4A), but also augmented the association of the upstream regulators of the RAS and PI3K signaling cascades with HCAR1 (Figure 3A), we investigated whether lactate-triggered signals indeed activated the two pathways. First, HCAR1 was knocked down in MDA-MB-231 cells using siRNAs, and the cells were stimulated with diluent 1 × PBS or 10 mM sodium lactate to investigate changes in the activation of the RAS and the PI3K pathways. While lactate treatment enhanced the phosphorylation of the RAS downstream effectors ERK1/2 and the PI3K downstream effector AKT as before (Figure 4A and Figure 6), downregulation of HCAR1 impaired their phosphorylation (Figure 6). Moreover, the levels of KRAS and GRB2 were also decreased after HCAR1 knockdown. Second, GRB2 is a downstream effector of growth factor receptor and GPCR and a signal-relaying hub-protein for both the RAS and the PI3K cascades [49,50,51,52]. We then knocked down GRB2, KRAS, or p110β in MDA-MB-231 cells to shut down the switch for both the RAS and the PI3K transduction pathways or the individual switch for either the RAS or the PI3K pathway and stimulated the cells with 1 × PBS or 10 mM sodium lactate as signaling input. The cell lysates were analyzed by Western blot for the levels of phosphorylated ERK or AKT as an indicator of RAS or PI3K pathway activation. We found that GRB2 knockdown potentiated KRAS levels and ERK1/2 phosphorylation but inhibited AKT phosphorylation (Figure 6). KRAS knockdown impaired ERK1/2 phosphorylation. PI3K knockdown, like GRB2 knockdown, increased ERK1/2 phosphorylation and decreased AKT phosphorylation. These data indicate that GRB2 mediates RAS and PI3K signaling in a counteracting way. Both the RAS and the PI3K pathways regulate lactate-triggered signaling, and downregulation of PI3K signaling potentiates RAS signaling.

## 4. Discussion 

Tumor tissues produce and consume lactate as a main metabolite and essential energy source. It was found that growing cancer cells consume more lactate than glucose [6,9,53]. Anticancer drug candidates targeting cell lactate-producing enzymes or plasma membrane lactate transporters are unsatisfactory [1,16,20], with challenges in drug delivery efficiencies and treatment complications. Inhibiting tumor progression by altering TME conditions has become a promising therapeutic strategy. The feasibility of this approach has been demonstrated in cell cultures and in animals, using LOX to deplete TME lactate or generate hydrogen peroxide (H_2_O_2_) or hydroxyl radicals [29,30,34,36,54]. It is plausible that, in addition to the cell-killing mechanisms mentioned above, LOX plays its cancer cell-inhibiting role by disrupting lactate and its associated cellular signaling, as examined in this study, or by other unknown mechanisms that warrant further study.

**Figure 7:**
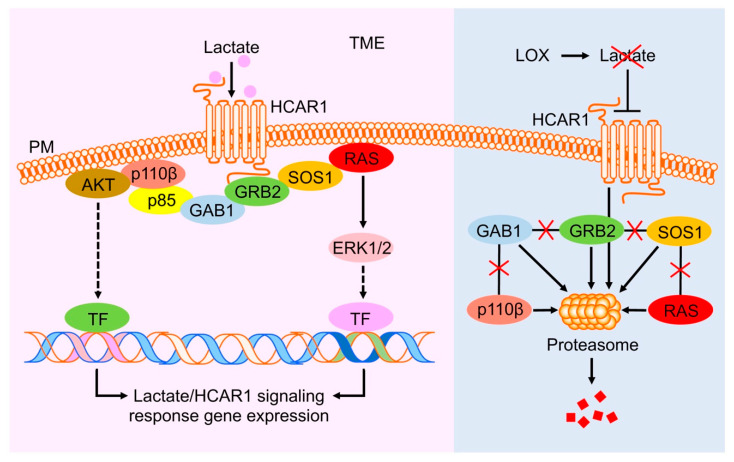
Model of lactate and LOX regulation of the HCAR1-RAS/PI3K signaling cascades. TME lactate activates HCAR1, which induces the assembly of a protein complex that includes the receptor itself, GRB2, SOS1, RAS, GAB1, p85, and p110β. Subsequently, the RAS and PI3K pathways are activated and transduce the lactate-triggered signals, via phosphorylated ERK1/2 and AKT, respectively, to the downstream transcription factors that further mediate the transcription of the genes involved in promoting the signaling and cancer cell survival and growth (left panel). Introducing LOX to the TME depletes lactate and renders HCAR1 inactive for triggering the assembly of the RAS and PI3K signaling complex. The disassembled HCAR1-associated RAS and PI3K signaling proteins are targeted for degradation by proteasomes (right panel). TME, tumor microenvironment; PM, plasma membrane; TF, transcription factor; the criss cross lines in the right panel represent removal or loss of interaction or signaling.

Lactate is a signaling ligand for HCAR1 [55]. The signaling cascades for cancer cell biological functions downstream from HCAR1 activation have not been defined. In this study, we found that lactate stimulated the association of GRB2 and the key RAS and PI3K signaling proteins with HCAR1 in a complex (Figure 3A). Interestingly, the levels of these HCAR1-associated proteins and their activation via the RAS or PI3K pathways, as indicated by ERK1/2 and AKT phosphorylation, were increased by lactate stimulation and decreased by LOX treatment (Figure 3C,D and Figure 4A). Importantly, LOX mediated the proteasomal degradation of HCAR1 and its signal-relaying proteins GRB2, SOS1, KRAS, GAB1, and p110β that are essential for the transduction of the RAS and PI3K pathways. These data suggest that lactate and LOX mediate the assembly and disassembly, respectively, of an HCAR1-associated intracellular protein complex governing the activation of RAS and PI3K downstream targets for oncogenic gene expression (Figure 7). This is a novel mechanism of TME lactate regulation of cellular functions that influences BCa cell survival and disease progression as modeled in our tumoroid 3D cultures (Figure 1 and Figure 2). Future work validating the regulation of this signaling nexus and its overlap or crosstalk with receptor tyrosine kinase (RTK) activation in oncogenesis will enhance our understanding about lactate-dependent cancer progression and present opportunities for the development of novel approaches to treat human cancers.

Our data showed that LOX depletion of lactate from the TME diminished the expression of cancer cell lactate transporters, MCT1 and MCT4 (Figure 4A). A decrease in these transporters can disrupt the exportation of lactate by lactate-producing cells and cause a shortage of lactate importation into the lactate-consuming cells, both of which are essential for cancer cell survival. Additionally, the pyruvate generated from the LOX and lactate reaction may form intermediate adducts with LOX before being catalyzed into acetic acid and carbon dioxide (CO_2_), and these intermediates can form even in a hypoxic environment [56], which is a common condition in tumors. The LOX-pyruvate adducts can affect pyruvate uptake and utilization as an energy source by cancer cells. Furthermore, it is possible that LOX depletion of lactate will speed up cancer cell glycolytic consumption of stored glucose and pyruvate while importing glucose from the TME is energy consuming. This internal drain of energy supplies may further stress the cancer cells. These drastic extracellular and intracellular changes induced by LOX not only alter the TME but may also cause cancer cell death.

## 5. Conclusions

LOX likely achieves the killing of cancer cells at multiple levels beyond its enzymatic role in lactate oxidation and H_2_O_2_ generation. The lactate-HCAR1-RAS/PI3K signaling cascades identified in this study are the tip of the iceberg of the potential for TME lactate regulation of cancer progression. Our data provides biologically relevant insights into reprogramming cancer cell metabolism and activities via manipulating the TME lactate levels and signaling. Targeting HCAR1 signaling upstream of the RAS and the PI3K oncogenic activation may be a feasible and effective anticancer therapeutic approach. 

## Data Availability

Data will be made available upon request from the corresponding author.

## Supplementary Materials

The following supporting information can be downloaded at: https://www.mdpi.com/article/10.3390/cancers16162817/s1, Figure S1: The sequence and expressing vector of TagRFP-T-LOX; Figure S2: Lactate increased the association of RAS and PI3K signaling pathway proteins with HCAR1 in T47D cells; Figure S3: Proteasome inhibition blocked LOX-induced degradation of HCAR1, its associated proteins, and the MCTs in T47D cells; Figure S4: Real-time PCR analysis of the expression of the genes corresponding to the key HCAR1-associated proteins in the RAS and PI3K signaling pathways as well as the lactate transporters.
