# Supplementary material for: Lactate Oxidase Disrupts Lactate-Activated RAS and PI3K Oncogenic Signaling
Source: Cancers (Basel). 2024 Aug 10;16(16):2817. doi: 10.3390/cancers16162817 (PMC11353192; doi:10.3390/cancers16162817)
Supplement: Supplementary file 1 [file cancers-16-02817-s001.zip › cancers-3127270-supplementary.pdf]

Supplementary Materials

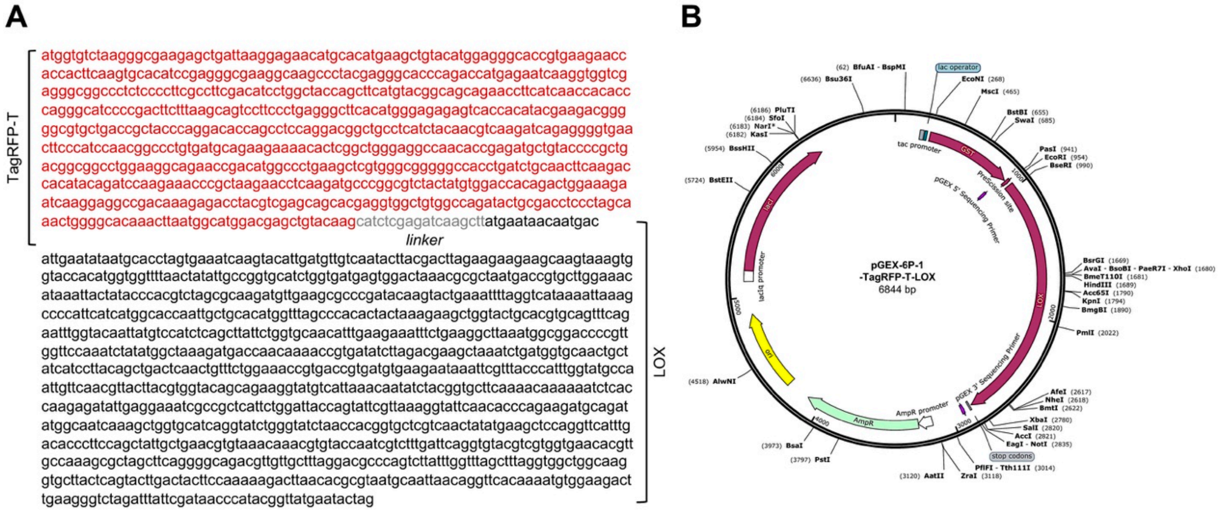

Figure. S1. The sequence and expressing vector of TagRFP-T-LOX. The TagRFP-T and LOX sequences were subcloned into the multiple cloning site between the EcoR1 and NotI restriction sites in the pGEX-6P-1 vector. The resulting pGEX-6P-1-TagRFP-T-LOX vector was expressed in the BL21 bacterial strain for TagRFP-T-LOX protein expression, affinity purification, and application in the experiment shown in Figure 1D.

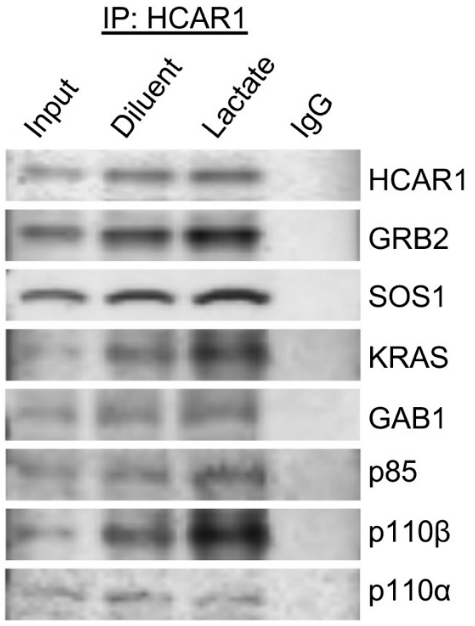

Figure. S2. Lactate increased the association of RAS and PI3K signaling pathway proteins with HCAR1 in T47D cells. IP of HCAR1 in T47D cell total protein lysates revealed the association of HCAR1 with RAS and PI3K signaling activators that was enhanced by lactate stimulation (Western blot).

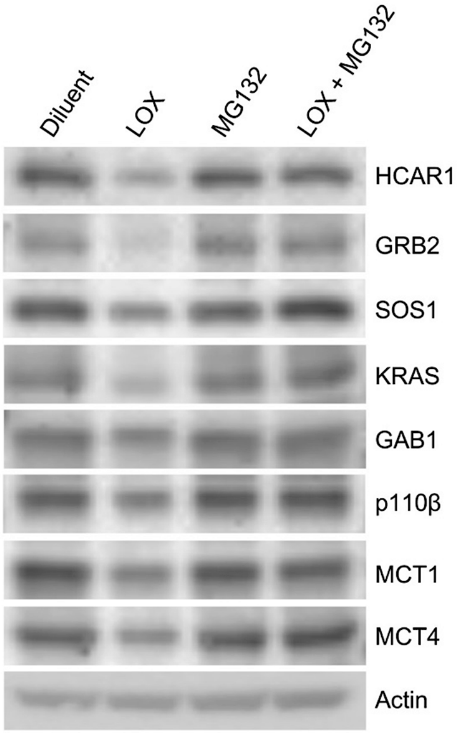

Figure. S3. Proteasome inhibition blocked LOX-induced degradation of HCAR1, its associated proteins, and the MCTs in T47D cells. Western blots of the protein expression in T47D cells after LOX or/and MG132 treatment for 24 hours. Diluent, 0.1 M sodium phosphate buffer; lactate, 10 mM; LOX, 0.05 U/ml.

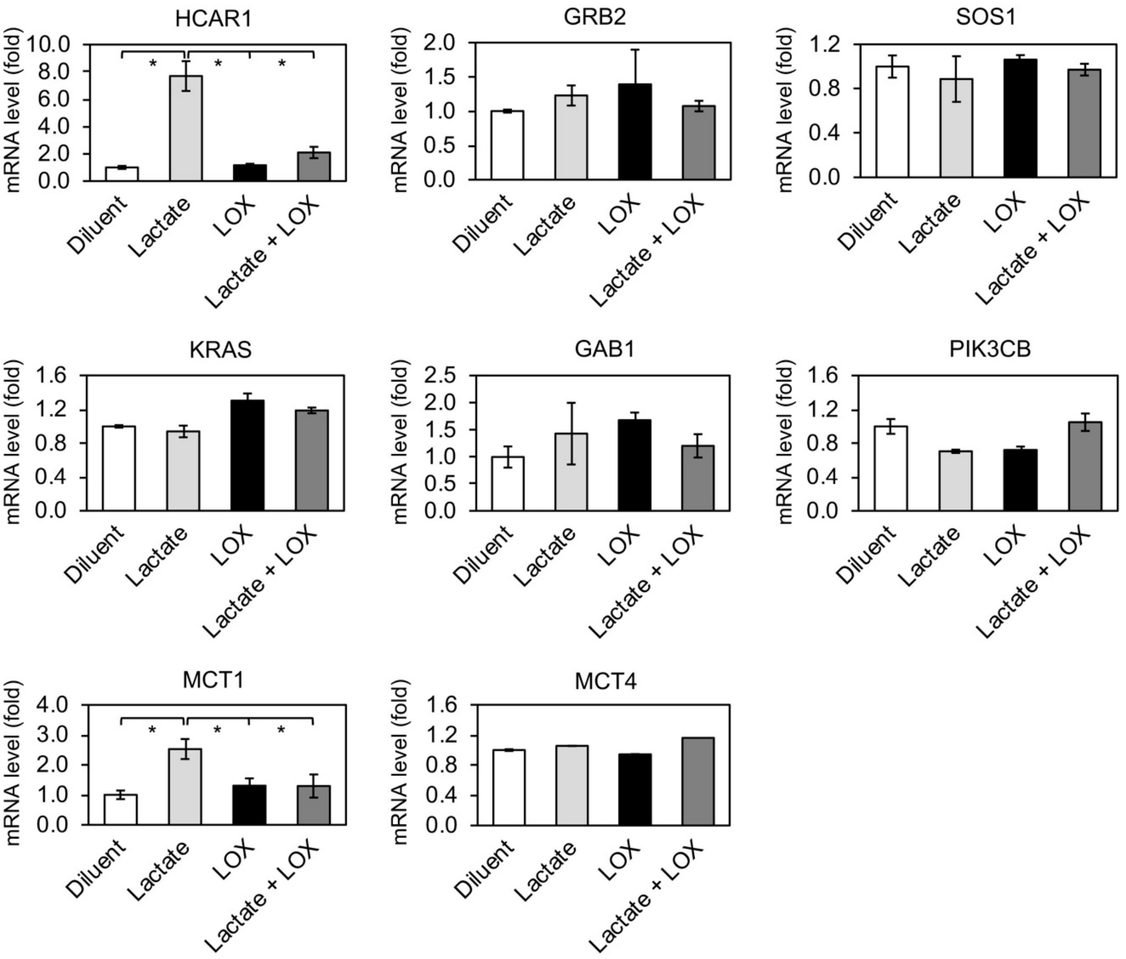

Figure. S4. Real-time PCR analysis of the expression of the genes corresponding to the key HCAR1-associated proteins in the RAS and PI3K signaling pathways as well as the lactate transporters. The fold change values were the target gene expression over actin beta (ACTB) expression levels and normalized to the diluent samples. Error bars, mean ± SD of 3 biological repeats, with each experimental condition having triplicate samples. * $p < 0.01$.
